# Supplementary material for: Evaluation of the basic assay performance of the GeneSoc® rapid PCR testing system for detection of severe acute respiratory syndrome coronavirus 2
Source: PLoS One. 2021 Mar 30;16(3):e0248397. doi: 10.1371/journal.pone.0248397 (PMC8009387; doi:10.1371/journal.pone.0248397)
Supplement: Attachment — Submitted filename: Comments PLOS ONE 20201224.docx [file pone.0248397.s001.docx]

This paper deals with the evaluation of a new PCR system, GeneSoc, for detection of SARS-CoV-2 from clinical specimens. The authors present a careful and thorough analysis of this system while comparing with another system, LightCycler 480, that is the major testing tool used in Japan. The aim addressed is interesting and important.

I would recommend it for acceptance after the minor points listed below.

Comments:

1. GeneSoc PCR system uses the primers and probes targeted to two genes (E and RdRp genes). Why these genes were chosen? Does the employment of these genes in this system have an advantage over LightCycler 480 system where one gene (N2 gene) is used as primer and probe?
2. As shown in Table 6, LightCycler 480 system was superior to GeneSoc system in term of the sensitivity for the detection of SARS-CoV-2. It is important not to miss the patients with a low viral load for preventing the spread of SARS-CoV-2 infection. Is there any room for improvement in the sensitivity of viral detection?
3. In specimen processing, the authors used a QIAamp Viral RNA Mini Kit. How long does it take to extract viral RNA? Is there a possibility of further time saving?
